# Real-time changes in brain activity during sacral neuromodulation for overactive bladder: evidence from functional near-infrared spectroscopy

**DOI:** 10.3389/fnins.2025.1436172

**Published:** 2025-04-28

**Authors:** Runtian Luo, Limin Liao

**Affiliations:** ^1^Department of Urology, China Rehabilitation Research Center, School of Rehabilitation of Capital Medical University, Beijing, China; ^2^China Rehabilitation Science Institute, Beijing, China

**Keywords:** sacral neuromodulation, urodynamics, overactive bladder, sacral neuromodulation (SNM), fNIRS (functional near infrared spectroscopy)

## Abstract

**Objective:**

The mechanisms underlying overactive bladder (OAB) and the role of sacral neuromodulation (SNM) in its treatment are not fully understood. In this study, functional near-infrared spectroscopy (fNIRS) was used to identify changes in brain activity during SNM in patients with OAB who responded to therapy.

**Methods:**

We employed a prospective trial method and idiopathic OAB patients selected for SNM treatment at our center were assigned to the experimental group and healthy adults matched by gender and age constituted the healthy control (HC) group. All participants completed 72-h urination diaries, the Overactive Bladder Symptom Score (OABSS), functional near-infrared spectroscopy (fNIRS) scans in both resting and task states, along with synchronous urodynamic monitoring. OAB patients were re-evaluated for these indicators after the SNM electrode implantation phase I test. The MATLAB toolbox NIRS-KIT was used to analyze and compare the differences in the internal functional connectivity (FC) of the prefrontal cortex (PFC) between the OAB group and the HC group before and after the treatment, in addition to assessing the differences in the PFC cortical activation/inactivation regions of the brain.

**Results:**

1. A total of 10 HCs and 12 patients with OAB were included. After the SNM Phase I test, 10 patients with OAB were successful, while 2 patients failed. The average frequency of 24-h urination, the levels of urgency and incontinence, and OABSS in the OAB group were significantly higher than those in the HC group. After the SNM treatment, the parameters recorded in urination diaries, OABSS, and urodynamic measures in the successful OAB group were significantly improved compared to their levels before treatment. 2. Task fNIRS results: Compared to the HC group, BA9 (left dorsolateral prefrontal lobe) was significantly inactivated in the successful OAB group before SNM treatment. Compared to the HC group, there was no significant difference in brain activation after SNM treatment in the successful OAB group. BA9 (left dorsolateral prefrontal lobe) and BA45 (the triangular part of the left inferior frontal gyrus) were significantly activated after the SNM treatment in the successful OAB group in contrast to their activation levels before treatment. 3. Resting fNIRS results: Compared to the HC group, the FC of the PFC in the successful OAB group was significantly weakened in both the empty and filled bladder states before SNM treatment; however, after SNM treatment, it returned to normal. Compared to the HC group, there was no significant difference in the FC of OAB patients in the empty bladder state after treatment, but the FC was significantly activated during the strong desire to void state.

**Conclusion:**

We found abnormal deactivation of the FC in the PFC and left dorsolateral prefrontal lobe (BA9), which might lead to socially inappropriate involuntary urination and could be a central pathogenesis of OAB. SNM restored the functional connectivity in the left dorsolateral frontal lobe, the triangular part of the left inferior frontal gyrus, and the interior of the prefrontal lobe in patients with OAB, which may be one of the central mechanisms of SNM treatment for OAB. Our results may provide valuable insights into the central pathogenesis of OAB and the central mechanism of SNM treatment.

## Introduction

Overactive bladder (OAB) is a syndrome characterized by urinary urgency symptoms, often accompanied by frequent urination and nocturia, with or without urgency urinary incontinence (UUI), in the absence of urinary tract infections or other obvious pathologies (Haylen et al., 2010). OAB has a very high incidence, ranging from approximately 16 to 43% (Irwin et al., 2006; Wang et al., 2011), which seriously affects patients’ quality of life. However, its pathogenesis remains unclear, which undoubtedly complicates treatment options. Sacral neuromodulation (SNM) can improve symptoms in patients with refractory OAB who do not respond to first-line therapies, including behavioral therapy and/or pharmacotherapy. However, the exact mechanism of action of SNM in the treatment of OAB remains unclear. Previous studies have suggested that possible mechanisms include inhibition of detrusor overactivity (DO) without affecting urethral resistance during micturition (Abrams et al., 2003), inhibition of the activity of C-fibers (Dmochowski and Gomelsky, 2011), and regulation of spinal cord reflexes and brain networks through peripheral afferents (van Voskuilen et al., 2006). In addition, some previous studies have reported that the areas activated or inactivated by the central nervous system above the spinal cord during the storage and urination stages are sorted according to the number of occurrences as follows: pons, periaqueductal gray matter, prefrontal cortex (PFC), cingulate gyrus, insula, cerebellum, thalamus, lentiform nucleus, hypothalamus, and premotor cortex (Griffiths and Tadic, 2008). However, they were cross-sectional studies and did not include a healthy control (HC) group. Therefore, it is impossible to determine to what extent the changes in brain activity OAB patients are due to other factors, and it is also unclear whether there is a difference in brain activity between OAB patients and healthy controls after SNM treatment. At present, the SNM stimulator can only be used for fMRI research when it is turned off (Elkelini and Hassouna, 2006), which may result in missing key, synchronous, and immediate information when it is turned on. Functional near-infrared spectroscopy (fNIRS) is a non-invasive brain imaging technology with a high temporal resolution that does not interfere with the operation of SNM, making it a better choice for studying the central mechanisms of SNM’s action (Leng and Chancellor, 2005; Elkelini et al., 2010). Therefore, we prospectively recruited an OAB group and an HC group and simultaneously performed urodynamic monitoring and fNIRS scanning during SNM stimulation, which may provide more comprehensive, reliable, and real-time information for exploring the central mechanism of SNM in OAB patients.

## Materials and methods

After approval from the Ethics Committee of the China Rehabilitation Research Center (IRB 2021-019-2), we recruited 12 patients with refractory idiopathic OAB who opted for SNM and 10 HCs matched for sex and age, all of whom provided informed consent. Patients diagnosed with OAB according to the criteria of the International Continence Society, with symptoms lasting for at least 3 months, were treated with SNM after the failure of at least two anticholinergic medications or a combination of an anticholinergic drug and a beta-3 agonist. The exclusion criteria included other causes of lower urinary tract symptoms (e.g., urinary tract infections, bladder stones or tumors, benign prostatic hyperplasia, pregnancy, and neurological diseases), history of SNM surgery, bladder pain syndrome, urinary retention (post-void residual volume > 150 mL), and pregnancy. The inclusion criteria for the HCs were a normal voiding diary, the OAB Symptom Score (OABSS), and urodynamic results. The exclusion criteria included neurological and urinary diseases, other major systemic conditions, and bladder dysfunction caused by oral medication.

### Experimental procedure

**Figure 1:**
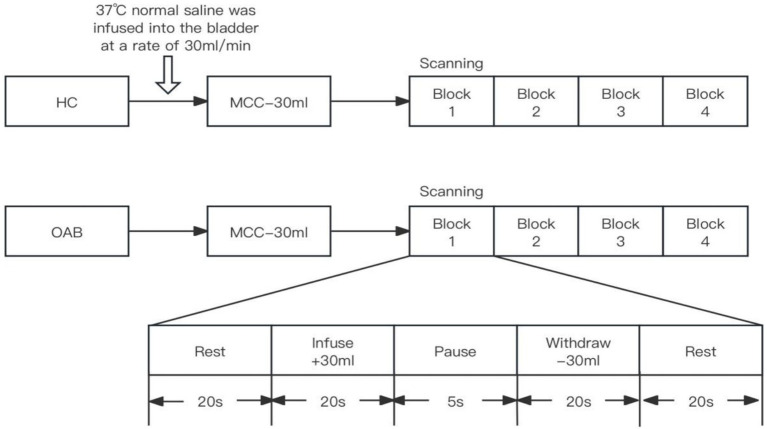
Process of the fNIRS scans synchronized with urodynamic monitoring and fNIRS equipment.

At baseline, all participants completed a 72-h voiding diary, the OABSS, and fNIRS scans with synchronous urodynamic monitoring. Subsequently, the patients with OAB received an electrode lead from the SNM system (InterStim II) along the third sacral nerve root using standardized surgical techniques guided by x-ray fluoroscopy. The SNM treatment was considered successful if at least one of the voiding diary parameters (i.e., mean frequency of urination per 24 h, mean urine volume, mean frequency of urinary urgency per 24 h, and mean frequency of urinary incontinence per 24 h) improved by at least 50% after 2–4 weeks of testing; otherwise, it was considered a failure. At this time, all patients with OAB were reevaluated and compared with the preoperative and HC groups. The specific process of fNIRS scans with synchronous urodynamic monitoring was as follows (Figure 1): (1) Urodynamic tests in this study were performed in accordance with the International Continence Society standards. The participant was asked to lie supine on the fNIRS scanning bed after urination, and a catheter was used to extract the residual urine. A double-lumen 7Fr catheter (for monitoring intravesical pressure and infusing and withdrawing water) and a single-lumen 10Fr rectal catheter (for monitoring abdominal pressure) were inserted into the urethra and rectum and connected to the urodynamic instrument. (2) The participant kept the eyes closed and wore earplugs. Then, the head was fixed to a matrix head coil with a sponge pillow and helmet. The lights in the room were dimmed to avoid acoustic and light stimuli, and the participant was asked to minimize head movements. The first fNIRS scan was performed. Then, the bladder was gradually filled with 37°C normal saline until the perfusion was stopped when the participant reported a strong desire to void, and a second fNIRS scan was performed. (3) After 50 mL was extracted from the bladder with a syringe, the repeated bladder infusion/withdrawal task began, which lasted 6 min and consisted of three blocks of five stages: rest, 20 s; infusion, 30 mL for 20 s; pause, 5 s; withdrawal, 30 mL for 20 s; and rest, 20 s. The block design task has clear advantages in reducing unnecessary data fluctuations and human interference. During the task, the fNIRS data were collected.

**Figure 2:**
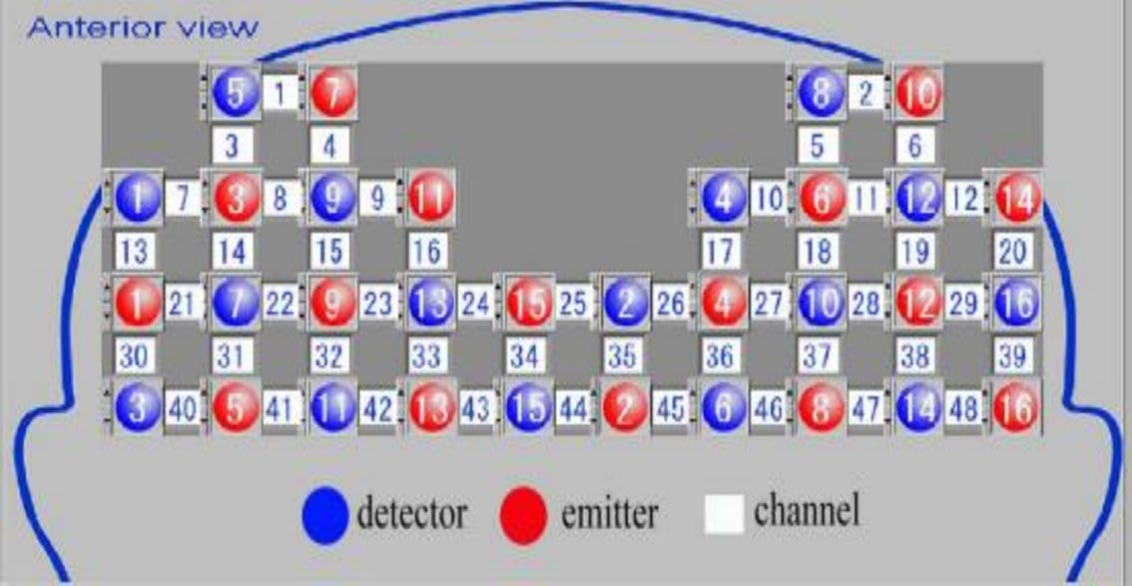
Layout of the light electrodes in the fNIRS acquisition instrument.

We selected the portable fNIRS scanner produced by the DaoJin Company. The probes were spaced 3 cm apart, with the lowest row of probes positioned along the Fp1-Fp2 line of the international 10–20 system (Figure 2). The functional transcranial brain atlas was used to obtain the position of all channels (Chs), including Montreal Neurological Institute (MNI) coordinates and the probability of associated brain regions from the Brodmann’s area (BA) atlas (Table 1).

**Table 1:** Ch locations for the fNIRS cap.

Ch	MNI coordinates (x, y, z)	BA	Brain area	Prob
1	34.84, −9.26, 69.78	6	Premotor and supplementary motor cortex	0.9
2	−42.88, −10.92, 63.07	3	Somatosensory cortex	1
3	47.77, −13.74, 61.09	3	Somatosensory cortex	1
4	38.29, 15.42, 58.52	6	Premotor and supplementary motor cortex	0.9
5	−43.63, 13.35, 53.61	6	Premotor and supplementary motor cortex	0.9
6	−53.42, −16.59, 56.15	2	Somatosensory cortex	1
7	56.89, −23.36, 52.98	9	Dorsolateral prefrontal cortex	0.71
8	50.52, 5.28, 50.87	6	Premotor and supplementary motor cortex	0.9
9	37.51, 29.64, 49.48	8	Includes frontal eye field	0.61
10	−42.72, 27.26, 44.72	8	Includes frontal eye field	0.61
11	−55.13, 2.24, 44.44	6	Premotor and supplementary motor cortex	0.9
12	−61.04, −26.06, 46.01	40	Supramarginal gyrus	0.71
13	64.65, −25.85, 41.39	40	Supramarginal gyrus	0.71
14	57.96, 4.06, 40.64	6	Premotor and supplementary motor cortex	0.9
15	46.1, 30.82, 39.25	8	Includes frontal eye field	0.61
16	28.13, 50.72, 36.07	8	Includes frontal eye field	0.61
17	−36.8, 46.73, 31.46	9	Dorsolateral prefrontal cortex	0.79
18	−51.78, 24.48, 33.06	9	Dorsolateral prefrontal cortex	0.79
19	−61.63, −2.81, 34.68	6	Premotor and supplementary motor cortex	0.9
20	−65.04, −29.62, 38.38	40	Supramarginal gyrus	0.71
21	63.37, −1.67, 30.79	6	Premotor and supplementary motor cortex	0.9
22	53.06, 28.62, 29.94	45	Inferior frontal gyrus, triangular part	0.79
23	40.07, 49.89, 25.65	9	Dorsolateral prefrontal cortex	0.79
24	20.2, 63.73, 23.77	9	Dorsolateral prefrontal cortex	0.79
25	−7.92, 66.05, 23.29	9	Dorsolateral prefrontal cortex	0.79
26	−26.4, 60.8, 21.4	9	Dorsolateral prefrontal cortex	0.79
27	−44.66, 45.28, 21.7	46	Dorsolateral prefrontal cortex	0.61
28	−55.98, 21.56, 24.68	9	Dorsolateral prefrontal cortex	0.79
29	−65.22, −7.35, 27.28	4	Primary motor cortex	0.98
30	67.04, −9.3, 17.27	3	Somatosensory cortex	1
31	59.3, 19.86, 19.5	9	Dorsolateral prefrontal cortex	0.79
32	48.7, 46.53, 12.36	46	Dorsolateral prefrontal cortex	0.61
33	29.74, 63.63, 13.27	10	Frontopolar area	0.92
34	9.37, 70.37, 12.07	10	Frontopolar area	0.92
35	−16.19, 68.4, 13.62	10	Frontopolar area	0.92
36	−38.43, 58.91, 9.29	10	Frontopolar area	0.92
37	−52.28, 39.05, 9.49	46	Dorsolateral prefrontal cortex	0.61
38	−60.88, 11.92, 14.38	44	Pars opercularis (Broca’s area)	0.73
39	−66.83, −14.27, 15.45	43	Subcentral area	0.68
40	62.97, 7.33, 8.98	6	Premotor and supplementary motor cortex	0.9
41	53.89, 39.99, 4.04	46	Dorsolateral prefrontal cortex	0.61
42	40.63, 60.05, 0.58	10	Frontopolar area	0.92
43	19.42, 70.09, 2.84	10	Frontopolar area	0.92
44	−8.28, 70.72, 0.82	10	Frontopolar area	0.92
45	−28.68, 64.9, 0.19	10	Frontopolar area	0.92
46	−44.19, 54.77, −2.08	10	Frontopolar area	0.92
47	−54.88, 34.76, 0.43	45	Pars triangularis	0.7
48	−63.53, −1.4, −2.08	22	Superior temporal gyrus	0.46

### Data acquisition

We used a MATLAB toolbox to perform data preprocessing, analysis, and final result visualization. The first and last 10 s of the fNIRS data were removed to ensure the stability of the signal. To eliminate slow time drifts, we applied a first-order detrend. We used the temporal derivative distribution repair method for motion correction. To remove artifacts, such as cardiac interference or respiration, the data were further filtered with a bandpass of 0.01–0.08 Hz. Previous studies have shown that the HbO signal is more sensitive than the HbR signal in reflecting local cerebral blood flow (CBF) fluctuations; therefore, we only analyzed changes in the HbO.

### Data analysis

Data comparison was performed using SPSS 21.0. A one sample *t*-test was used to compare the inner group differences, and a two sample *t*-test was used to compare the group differences.

## Results

### Comparison of the demographic and clinical characteristics between the HC and OAB groups

A total of 10 healthy individuals and 12 patients with OAB were enrolled. After the SNM phase I treatment, 10 patients with OAB were successfully treated, while two patients did not respond to the treatment. There were no significant differences in age, gender, years of education, handedness, and the score of a strong desire to void between the HC group and OAB groups, as shown in Table 2 (*p* > 0.05). The 24-h mean frequency of urination, mean frequency of urinary urgency, mean frequency of urinary incontinence, and OABSS in the OAB group were significantly higher than those in the HC group. Moreover, these indicators were significantly reduced after the SNM treatment in the successful group, and the differences were statistically significant (*p* < 0.05). In addition, the mean urine volume, FSV, and MCC of the OAB group were significantly lower than those of the HC group before the treatment and were significantly increased after the SNM treatment in the successful group. The differences were statistically significant (*p* < 0.05). Urodynamic monitoring of seven patients in the successful OAB group suggested DO, which was significantly improved after the SNM treatment (*p* < 0.01).

**Table 2:** Comparison of the general clinical data between the HC group and the OAB group.

	HC	Preop OAB	Postop OAB	*p*-value (HC/Preop OAB)	*p*-value (HC/Postop OAB)
Total no.	10	12	12		
Mean age (mean ± SD)	40.69 ± 15.19	49.22 ± 16.35	49.22 ± 16.35	0.126	
No. sex (%)				0.746	
Male (%)	4(40%)	4(33%)	4(33%)		
Female (%)	6(60%)	8(67%)	8(67%)		
Mean yrs. of education (mean ± SD)	10.50 ± 3.14	11.06 ± 3.04	11.06 ± 3.04	0.604	
Handedness	Rt-handed	Rt-handed	Rt-handed		
72-h voiding diary					
Median frequency of urination/24 h (range)	6(5–7)	14.37(5–114)	7.33(5–21)	<0.001	< 0.001
Mean mL urine volume (mean ± SD)	356.24 ± 36.63	125.30 ± 76.34	205.03 ± 77.62	<0.001	<0.001
Median frequency of urinary incontinence/24 h (range)	0(0–0)	0(0–6)	0(0–2)	0.004	0.022
Median frequency of urinary urgency/24 h (range)	0(0–0)	8(4–123)	4(1–20)	<0.001	<0.001
Median OABSS (range)	0(0–0)	10(1–12)	6(2–10)	<0.001	<0.001
Post-void residual (mL)	<10 mL	<10 mL	<10 mL		
Urodynamic monitoring					
mL FSV (mean ± SD)	215.34 ± 43.57	128.50 ± 63.78	152.41 ± 61.29	<0.001	0.003
mL MCC (mean ± SD)	430.88 ± 77	235.89 ± 96.25	264.55 ± 85.63	<0.001	0.005
Median cm H_2_O max Pdet during DO (range)		32(17–95)	20(2–92)		0.007
Median cm H_2_O Pdet at end of filling (mean ± SD)	2.21 ± 1.43	4 ± 2.9	2.56 ± 2.12	0.079	0.181

### Comparison of the resting fNIRS data between the OAB and HC groups in ‘empty bladder and strong desire to void’ states

#### Comparison between the successful OAB group and the HC group before the SNM treatment

**Figure 3:**
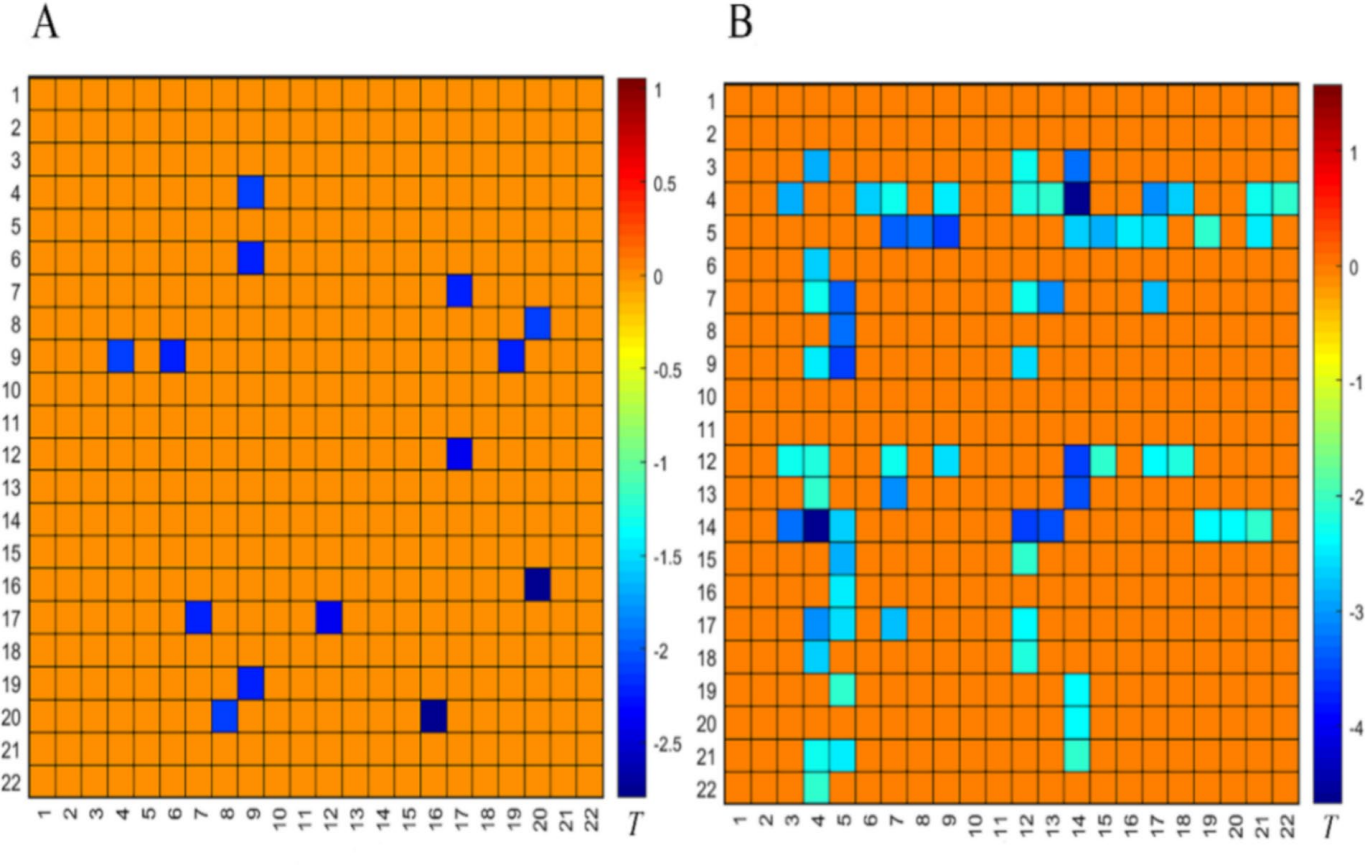
Comparison of the internal FC matrix of the prefrontal cortex in the empty bladder state **(A)** and the strong desire to void state **(B)** between the successful OAB group and the HC group before the SNM treatment. The color bar represents the T value, with cold colors indicating the FC weakening and warm colors indicating the FC enhancement.

Before the SNM treatment, compared to the HC group, the FC in the PFC of the successful OAB group was significantly deactivated in the empty bladder state, as shown in Figure 3A (*p* < 0.05). The following three BAs were involved: BA9 (right and left dorsolateral prefrontal lobes), BA10 (left frontal pole, right frontal pole), and BA46 (right and left dorsolateral prefrontal lobes). Compared to the HC group, the FC in the PFC of the successful OAB group was significantly deactivated in the strong desire to void before the SNM treatment, as shown in Figure 3B (*p* < 0.05). The following five BAs were involved: BA9 (right and left dorsolateral prefrontal lobes), BA10 (left frontal pole, right frontal pole), BA44 (left insula of Broca’s area 5), BA45 (left inferior frontal gyrus, triangular part), and BA46 (right and left dorsolateral prefrontal lobes).

#### Comparison between the successful OAB group and the HC group after the SNM treatment

**Figure 4:**
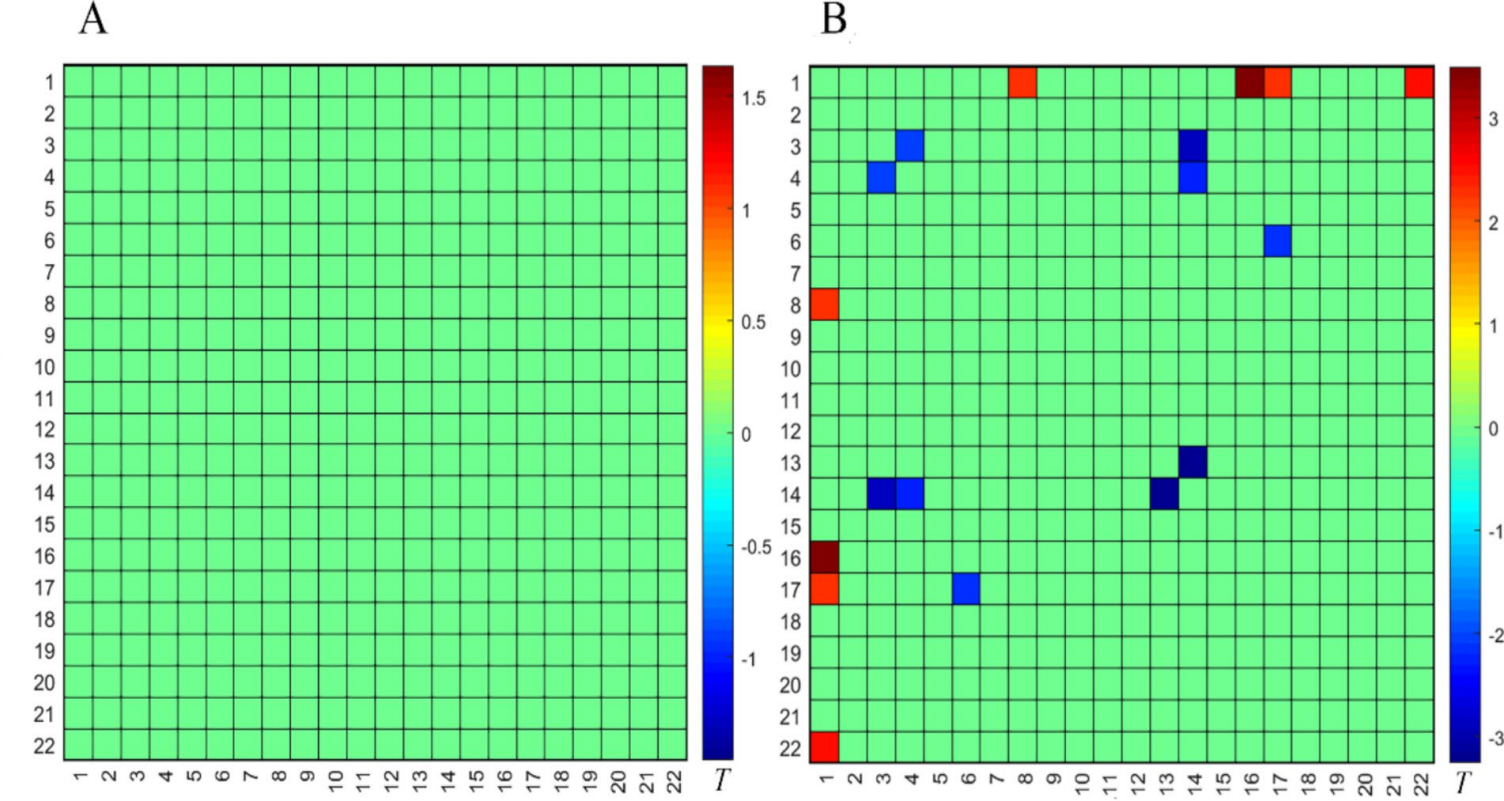
Comparison of the FC matrix of the prefrontal cortex in the empty bladder state **(A)** and the strong desire to void state **(B)** between the successful OAB group and the HC group after the SNM treatment. The color bar represents the T value, with cold colors indicating the FC weakening and warm colors indicating the FC enhancement.

Compared to the HC group, there was no significant difference in the FC of the PFC during the empty bladder state in the successful OAB group after the SNM treatment (Figure 4A). However, the FC of the PFC was significantly activated during the strong desire to void (Figure 4B). The following four BAs were involved: BA9 (right dorsolateral prefrontal lobe), BA10 (right frontal pole), BA45 (left inferior frontal gyrus, triangular part), and BA46 (right dorsolateral prefrontal lobe). The significant deactivation of the FC in the PFC involved three BAs: BA9 (left and right dorsomedial prefrontal lobes), BA10 (right frontal pole), and BA46 (left dorsomedial prefrontal lobe).

#### Comparison between the pre-treatment and post-treatment SNM in the patients with OAB in the successful group

**Figure 5:**
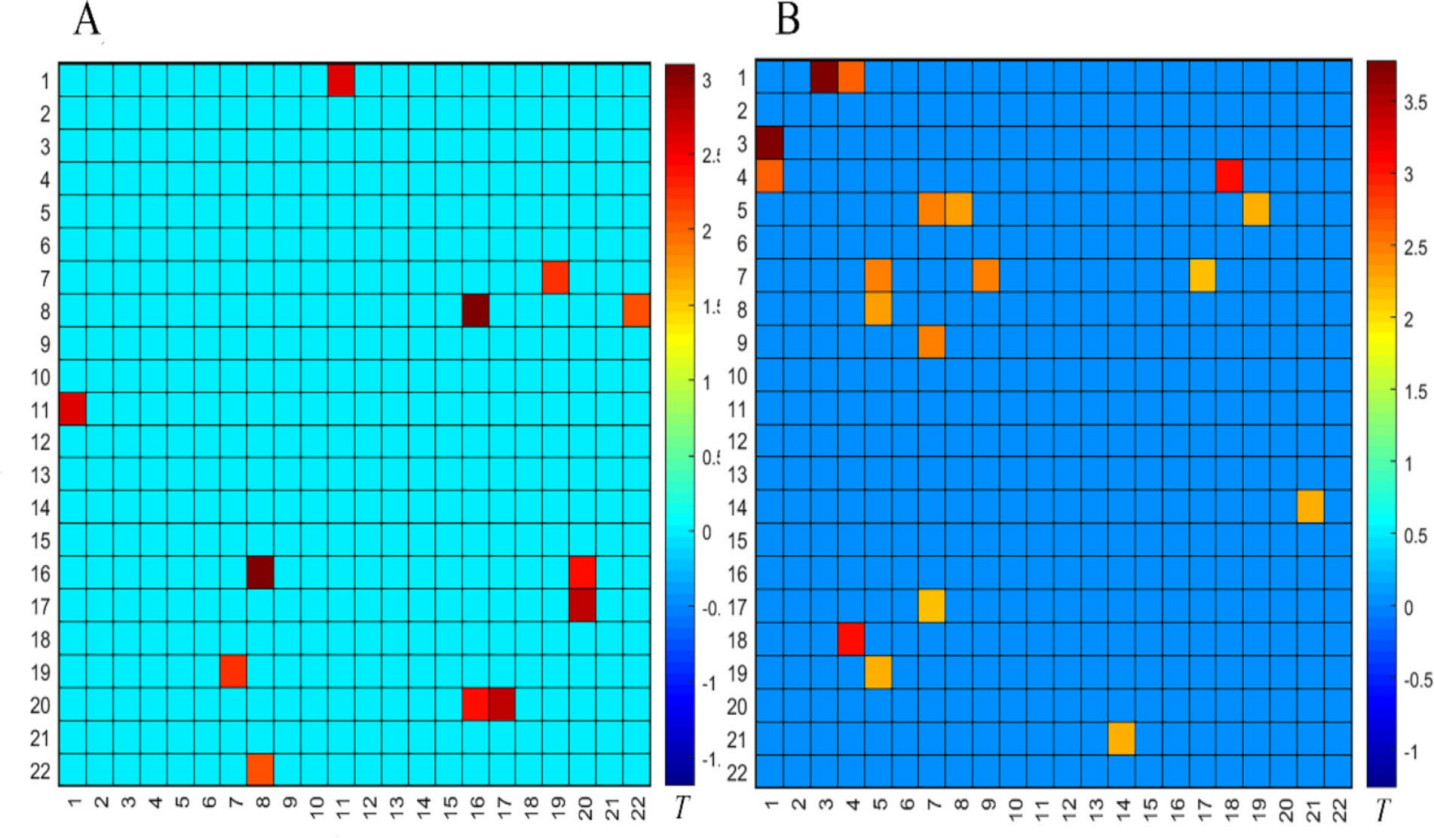
Comparison of the FC matrix of the prefrontal cortex in the empty bladder state **(A)** and the strong desire to void state **(B)** before and after the SNM treatment in the successful OAB group. The color bar represents the T value, with cold colors indicating the FC weakening and warm colors indicating the FC enhancement.

Compared to the pre-treatment SNM in the successful OAB group, the FC in the PFC of the successful OAB group after the SNM treatment was significantly activated during the empty bladder state, as shown in Figure 5A (*p* < 0.05). This activation involved four BAs: BA9 (left and right dorsolateral prefrontal lobes), BA10 (left frontal pole, right frontal pole), BA45 (left inferior frontal gyrus, triangular part), and BA46 (right dorsolateral prefrontal lobe). Compared to the pre-treatment SNM in the successful OAB group, the FC in the PFC during the strong desire to void was significantly activated after SNM treatment, as shown in Figure 5B (*p* < 0.05). This activation involved three BAs: BA9 (left dorsomedial prefrontal lobe, right dorsomedial prefrontal lobe, left dorsomedial prefrontal lobe, right dorsomedial prefrontal lobe), BA10 (left frontal pole, right frontal pole), and BA46 (left dorsomedial prefrontal lobe, right dorsomedial prefrontal lobe).

### Comparison of the task fNIRS data between the OAB and HC groups in the ‘empty bladder and strong desire to void’ states

**Figure 6:**
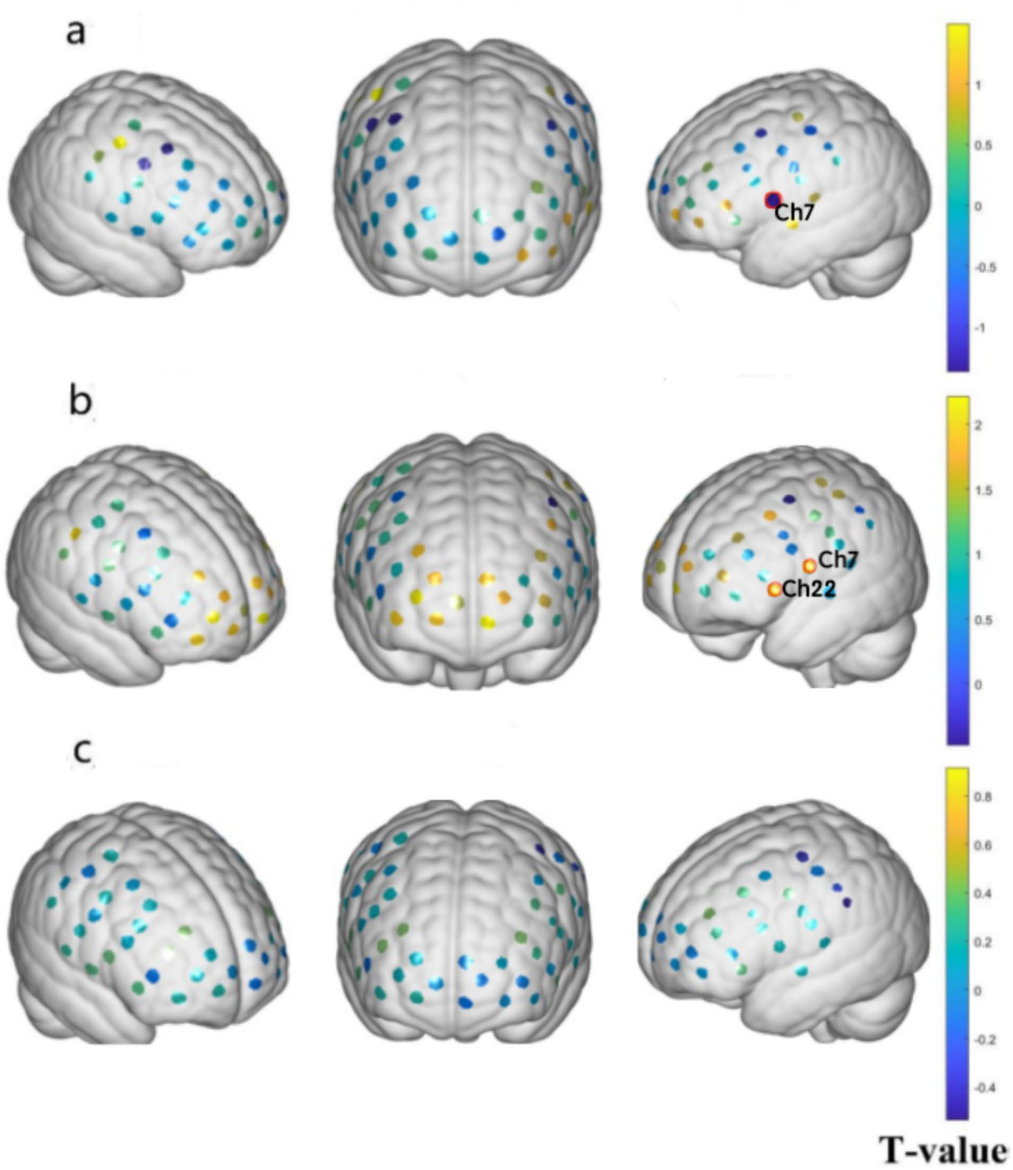
Prefrontal activation changes in the successful OAB group and the HC group before and after the SNM surgery. **(A)** The brain regions activated in the successful OAB group before the SNM treatment compared to the HC group. **(B)** Compared to the preoperative successful OAB group, the brain regions successfully activated in the postoperative OAB group; **(C)** Compared to the HC group, the brain regions activated after the SNM treatment in the successful OAB group. Channels marked with red circles indicate significant differences in activation (*p* < 0.05, FDR corrected). The color bar represents group-level T values. Warm colors indicate activation, and cold colors indicate deactivation.

Compared to the HC group, the successful OAB group before the SNM treatment showed significant deactivation in BA9, as shown in Figure 6A (left dorsolateral prefrontal lobe) (*p* < 0.05). Compared to the HC group, there was no significant difference in brain activation after the SNM treatment in the successful OAB group, as shown in Figure 6C (*p* > 0.05). After the SNM treatment, in the successful OAB group, BA9 (left dorsolateral prefrontal lobe) and BA45 (left inferior frontal gyrus, triangular part) were significantly reactivated compared to before the treatment, as shown in Figure 6B (*p* < 0.05).

## Discussion

fNIRS is a non-invasive optical neuroimaging technology, which is similar to fMRI brain functional imaging. Compared to fMRI, fNIRS has a higher sampling rate (up to 100 Hz), lower cost, and can more accurately characterize the changes in blood oxygen signals (Tong et al., 2011).

In this study, the parameters from the 72-h voiding diary, FSF, and MCC of the preoperative OAB group improved significantly after the SNM treatment. At the same time, the DLPFC, which is involved in bladder sensation, was significantly activated in the postoperative OAB group. We believed that the central mechanism of action of SNM was to correct abnormal bladder afferent signals by regulating ascending sensory nerve impulses. Moreover, the maximum Pdet during DO in the preoperative OAB group was significantly reduced after the SNM treatment. This finding is consistent with those of a previous study, suggesting that SNM could also improve lower urinary tract symptoms by inhibiting DO (Groen et al., 2006).

According to the results of the resting fNIRS, we found that, compared to the HC group, the FC in the PFC of the successful OAB group was significantly deactivated in the empty bladder state before the SNM treatment and returned to normal after the SNM treatment. Therefore, we believe that SNM can improve the cooperation between different brain regions within the PFC of patients with OAB, bringing it closer to normal levels, thus improving lower urinary tract symptoms in these patients.

According to the results of the task fNIRS, we found that, compared to the HC group, BA9 (left dorsolateral prefrontal lobe) was significantly inactivated in the successful OAB group before the SNM treatment. After the SNM treatment, BA9 (left dorsolateral prefrontal lobe) and BA45 (left inferior frontal gyrus, triangular part) were significantly reactivated. The PFC is involved in advanced cognition, language, emotional processing, and social interaction, all of which are crucial aspects of human executive function. The dorsolateral frontal lobe area is mainly responsible for executive function (Teffer and Semendeferi, 2012). Previous research has found through animal experiments and clinical observations that multiple brain regions, including the prefrontal cortex, are involved in bladder voiding control (Thor and de Groat, 2010). Blok et al. used PET to study brain activity changes in healthy men during bladder filling, urination, and after urination. They observed that blood flow in the right inferior frontal gyrus, right anterior cingulate gyrus, thalamus, and periaqueductal gray matter changed after bladder filling and urination (Blok et al., 1997). Tadic et al. (2012) studied the brain activity of elderly women with OAB, which leads to impaired urination control, through brain fMRI combined with synchronous urodynamic detection. The results showed that the auxiliary motor area, superior frontal gyrus, and dorsolateral prefrontal lobe of the brain were activated, while the ventromedial prefrontal lobe was deactivated in older patients with OAB during urinary urgency. In the subgroup of patients prone to DO, the activation of the auxiliary motor area was stronger, suggesting that there is a compensatory response when the normal urination control mechanism fails. Sakakibara et al. (2010) used fNIRS to detect changes in oxy-Hb in the prefrontal lobe during natural and continuous bladder filling, as well as sitting urination, in healthy adults and patients with OAB. The study showed that, during the natural bladder filling, the bilateral lateral prefrontal lobes (especially Brodmann areas 8, 10, and 46) were activated. Among them, the activation of the frontal lobes in the OAB group was weaker than that in the healthy control group, and oxy-Hb in the bilateral frontal lobes continued to decrease during urination. This suggests that the bilateral prefrontal lobes play an important role in the brain’s bladder control mechanism. Zuo et al. observed in an imaging study of healthy individuals with normal detrusor function and patients with incontinence due to DO during the filling phase that the inferior frontal gyrus and the middle temporal gyrus play a role in the brain’s control of urinary bladder storage. They believed that the inferior frontal gyrus is related to the inhibition of detrusor contraction (Zuo et al., 2019). Researchers also found that white matter injury in the medial prefrontal lobe can affect bladder function long-term, while gray matter injury in the medial frontal lobe can lead to urinary incontinence (Andrew and Nathan, 1964). The inferior frontal gyrus anatomically belongs to the ventromedial frontal lobe. The ventromedial frontal lobe is involved in decision-making within certain emotional and social contexts and has extensive connections with the limbic system, hypothalamus, amygdala, anterior cingulate cortex, and other regions.

This study has some limitations. Due to the small sample size, the results may be biased. In addition, because of the limited detection depth, fNIRS can only detect activity on the surface of the cerebral cortex and not in the deep brain nuclei. In the future, we aim to increase the detection depth by combining fNIRS with other techniques.

## Conclusion

We found abnormal deactivation of the FC in the PFC and left dorsolateral prefrontal lobe (BA9), which may contribute to socially inappropriate involuntary urination and could be a central pathogenesis of OAB. SNM restored the functional connectivity of the left dorsolateral frontal lobe, the triangular part of the left inferior frontal gyrus, and the interior of the prefrontal lobe in patients with OAB. This restoration may be one of the central mechanisms behind the effectiveness of SNM treatment for OAB. Our results may provide valuable insights into the central pathogenesis of OAB and the underlying mechanisms of SNM treatment.

## Data Availability

The original contributions presented in the study are included in the article/supplementary material, further inquiries can be directed to the corresponding author.
